# Professional reasoning of occupational therapy driver rehabilitation interventions

**DOI:** 10.1111/1440-1630.12804

**Published:** 2022-05-16

**Authors:** Angela Berndt, Claire Hutchinson, Dillon Tepper, Stacey George

**Affiliations:** ^1^ Allied Health & Human Performance University of South Australia Adelaide SA Australia; ^2^ Caring Futures Institute, College of Nursing and Health Sciences Flinders University Bedford Park SA Australia; ^3^ Integrated Mental Health Services Mount Gambier Hospital Mount Gambier SA Australia; ^4^ Allied Health, Northern Adelaide Local Health Network Lyell McEwin Hospital Elizabeth Vale SA Australia

**Keywords:** activities of daily living, automobile driving, clinical reasoning, occupational therapy, qualitative research

## Abstract

**Introduction:**

Driver‐trained occupational therapists are advanced practitioners who work with people to help maintain their independence and autonomy through driving. There is a lack of investigation of professional reasoning processes for why interventions are recommended by driver‐trained occupational therapists. This research project sought to explore the reasoning of driver‐trained occupational therapists when they plan, implement, and reflect on driver rehabilitation interventions.

**Methods:**

In‐depth semistructured interviews (*n* = 7) and one focus group (*n* = 5) were conducted with 12 experienced driver‐trained occupational therapists, comprising a wide range of experience, client populations, and licensing jurisdictions. Data were analysed using a modified template analysis approach.

**Results:**

Seven higher order modes that reflect professional reasoning theory and hierarchical models were evident in the work of the driver‐trained occupational therapists, with no new modes of reasoning emerging. Ethical reasoning regarding the balance of safety versus client independence was an overarching shared framework, with therapists mostly using interactive and conditional reasoning in practice. Twenty‐three second‐level themes were identified that exemplify how the reasoning modes operate in practice. Therapists described assessment activity even when solely asked about intervention, indicating the importance of assessment to intervention design. The full hierarchy of reasoning was evident during the rehabilitation phase.

**Conclusion:**

These findings elucidate the application of professional reasoning in advanced occupational therapy practices and could support driver‐trained occupational therapists in making driving rehabilitation recommendations if used in reflective practices.

**Key Points for Occupational Therapy**

- In prescribing driver rehabilitation, driver‐trained occupational therapists demonstrate a commitment to ethical professional reasoning as an overarching practice.
- Driver‐trained occupational therapist integrate all types of professional reasoning into their practice, in a nonlinear manner.
- The complex reasoning identified confirms occupational therapy driver rehabilitation as an advanced scope of practice.

## 1 INTRODUCTION

Occupational therapy driver assessors are trained to provide assessment, intervention, and recommendations related to functional ability to drive. Their role and recommendations can have major implications on an individual's return to driving and community safety. Safe and effective rehabilitation interventions and driving outcomes rely on well‐developed clinical and professional reasoning skills. Driver‐trained occupational therapists are characteristically advanced practitioners requiring postgraduate training given such work requires complex professional decision making in a higher risk, highly skilled area of practice. Research into driver rehabilitation interventions primarily consists of the effectiveness of car adaptations and modifications (Lowe et al., 2014; Unsworth & Baker, 2014), computer‐based driving simulator training (George et al., 2014), off‐road training (Crotty & George, 2009; Frith et al., 2017; Ross et al., 2018), on‐road training (Golisz, 2014; Unsworth & Baker, 2014), personal adaptive devices (Golisz, 2014; Wood et al., 2016), physical fitness programmes (Gaudet et al., 2016; Golisz, 2014), and psychosocial support (Golisz, 2014). There is a scarcity of research exploring the clinical reasoning processes for why interventions are recommended from a driver‐trained occupational therapist perspective.

Clinical reasoning is a multifaceted process inherent to many health professions requiring purposeful, goal‐focused cognitive, meta‐cognitive, and clinical skills within differing contexts (Young et al., 2020). Regardless of context, occupational therapists use clinical reasoning to plan, provide, and reflect on the process and effectiveness of client service provision (Higgs et al., 2019; Schell & Schell, 2018; Unsworth & Baker, 2016). In practice, clinical reasoning involves applying modes of thinking that underpin how the therapist understands people's needs, defines problems and informs therapeutic decision‐making (Schell & Schell, 2018; Unsworth & Baker, 2016).

It is theorised that occupational therapists use distinct yet overlapping or integrated modes of reasoning. Early individual modes identified, namely, procedural, interactive, and conditional (Fleming, 1991), have been supplemented by scientific, narrative, pragmatic, and ethical reasoning (O'Brien, 2018; Higgs et al., 2019; Lysaght & Bent, 2005; Unsworth & Baker, 2016). However, in the multidisciplinary context, there is disparity in language with a scoping review identifying that whilst 37% of 625 papers used the term clinical reasoning, another 110 terms were also used (Young et al., 2020). This review (Young et al., 2020) recommends authors therefore make their intended meaning of clinical reasoning explicit. Due to the integrative nature of reasoning in practice, the Hierarchical Model of Professional Reasoning (Unsworth, 2004, 2017), which draws on prior literature, was adopted for this study. In this model, the therapist's worldview (assumptions, values, beliefs) and ethical reasoning provide a meta‐cognitive frame in which the other forms of reasoning are processed, in a therapeutic encounter and client centred environment (Unsworth, 2017). Ethical reasoning is the process through which actions and judgments are based on a set of principles (Kyler‐Hutchison, 1988), contemplating the moral dimensions of the situation (Cole & Creek, 2016) to identify risk, generate alternative solutions and actions (Schell, 2019). Within the hierarchy, each type of reasoning is distinct yet overlaps with other types as the therapist analyses and responds to client need (Unsworth, 2017).

Procedural reasoning occurs when, through the “procedures” of therapy, the practitioner focuses on the clients' disease or disability, determining appropriate modalities to improve performance through identifying the problem, goal setting, and treatment planning (Fleming, 1991; Schell, 2019; Unsworth, 2017). Sinclair (cited in Cole & Creek, 2016) describes reasoning processes as *evidence discovery*, including gathering data, completing assessments, identifying potential problems, recognising cues and defining focus of intervention. Interactive reasoning involves getting to know clients and their subjective experiences (Fleming, 1991; Mattingly & Fleming, 1994) to build positive interpersonal relationships, and collaborative problem solving (Schell, 2019).

Scientific reasoning processes supplement this discovery phase when the therapist methodically gathers evidence to generate and test hypotheses (Schell, 2019). Narrative reasoning uncovers a client's occupational story and how they make sense of events (Mattingly, 1991). Similarly, occupational therapists create narratives, or collaborative stories, about clients' experiences and roles, to better understand their work through concepts (Mattingly, 1991; Schell, 2019; Unsworth, 2017). Conditional reasoning is a rapid synthesis of information elicited, within the client's temporal and social context, that draws on the therapists past experiences to determine the best course of action (Cole & Creek, 2016). Decisions are instilled with lessons learned through trial and error (Fleming, 1991; Mattingly & Fleming, 1994) and theory application (Cole & Creek, 2016), where the therapist can “see” multiple futures for the client (Schell, 2019). Dependent on role and context, therapists develop different levels of skill in reasoning modes, remaining at a novice level in some modes while demonstrating expertise in others (Cole & Creek, 2016). Pragmatic reasoning considers environmental, political, and economic factors within the service context (Schell, 2019; Unsworth, 2017) to fit therapy possibilities within current realties of service provision (Schell, 2019). Pragmatic reasoning can also include the personal context of the therapist's motivation, skills, and knowledge (Unsworth, 2017).

In 2019, a scoping review of occupational therapy clinical reasoning evidence (1982 to 2017) identified three areas of research: student reasoning, theoretical aspects, and specific professional fields, for example, mental health or spinal cord injury, with a subset of 18 publications in modalities of reasoning (Marquez‐Alvarez et al., 2019). The review located 208 articles, none of which focussed on driver rehabilitation interventions. However, Unsworth, a driver assessment focused researcher, was identified as the largest sole contributor to professional reasoning research (Marquez‐Alvarez et al., 2019). For example, in a single case study design, via analysis of head mounted video footage, a driver‐trained occupational therapist was able to articulate a variety of reasoning modes, with procedural dominating due to being in the assessment phase of service provision (Unsworth, 2001). Research suggests that a shift in scope may lead the driver rehabilitation occupational therapist to a more novice stage of reasoning before past and present skills reintegrate into the advanced scope. Novice practitioners or advanced beginners are said to use procedural and scientific reasoning by preference, building up to the higher order forms of reasoning, such as conditional, with experience (Clifford O'Brien, 2018). Clinical expertise is a pillar of evidence‐based practice (Hoffman et al., 2010), and professional reasoning is an established theoretical and conceptual framework that help articulate expertise and support the transition from novice to expert practitioner in a field that requires high autonomy and decision making. This study sought to articulate the reasoning processes driver‐trained occupational therapists used when they develop and implement rehabilitation interventions.

## 2 METHODS

Ethics approval for this study was provided by the [University blinded for peer review, University of South Australia] Human Research Ethics Committee (201969).

### 2.1 Recruitment and eligibility

Driver‐trained occupational therapists were recruited from Occupational Therapy Australia Driver Interest Groups using website advertisements, and via Facebook posts on driver rehabilitation occupational therapy groups nationally. All advertisements were accompanied with an information sheet. To be eligible participants needed to be English‐speaking driver‐trained occupational therapists practicing in Australia.

### 2.2 Participants

Twelve participants took part in this study, 11 females and one male. Experience ranged from 4 to 37 years (Mean = 23.2, SD = 11.7). Postgraduate driver training experience ranged from <1 to 32 years (Mean = 10.8, SD = 9.7). Seven (58%) participants worked in private practice, four (33%) worked in the public sector and one (8%) worked in both. Participants worked across six Australian states (Table 1). Purposive sampling was used (Creswell & Clark, 2017) to gather a wide variety of experience, driver rehabilitation interventions, and work environments.

**TABLE 1:** Participant Information

	Frequency (%)
Gender	
Female	11 (92%)
Male	1 (8%)
Occupational therapy experience	
Less than 5 years	1 (8%)
5 to 10 years	‐
11 to 20 years	5 (42%)
21 to 30 years	1 (8%)
Over 31 years	5 (42%)
Driver‐training experience	
Less than 5 years	5 (42%)
5 to 10 years	1 (8%)
10 to 20 years	5 (42%)
20 to 30 years	‐
Sector	1 (8%)
Public	3 (25%)
Private	8 (67%)
Both	1 (8%)
States & territory^a^	
ACT	‐
NT	6
NSW	5
QLD	1
SA	10
TAS	‐
VIC	2
WA	1

^a^ Six participants working in one state, six participants in multiple states.

### 2.3 Materials

A schedule of key questions/prompts was developed from relevant occupational therapy and clinical reasoning literature. The interview schedule (Table 2) was piloted with two experienced therapists, and explored the impact of client factors, use of theoretical frameworks, research evidence and guidelines, decision making processes, how risks and benefits were weighed up, and how therapists measured intervention outcomes.

**TABLE 2:** Interview or focus group questions

How does a client's reported subjective experience of an illness or injury influence the implementation of [intervention/s]?
Tell me about shared decision‐making with your clients.
How do your client's life roles and desired activities impact [intervention/s]?
How does the sensation of just being around your client, in person, influence interventions?
How does your client's story, or who they are as a person, impact your implementation of [intervention/s]?
What explanations or descriptions are used to convey how the implementation of [intervention/s] is going?
How does the nature of a client's illness or injury impact upon your decision to implement [intervention/s]?
Tell me about the theoretical frameworks, research evidence (*added*) and guidelines that underpin the implementation of [intervention/s].
What potential occupational performance problems associated with [condition/s] provide a rationale for the implementation of [intervention/s]?
Why is the implementation of [intervention/s] part of your therapy routine or regimen?
What are the facilitators and constraints to implementing [intervention/s]? (pragmatic)
What considerations of the environment do you have when developing or implementing [intervention/s]? (pragmatic)
How do you weigh up the risks and benefits of implementing [intervention/s]?
How do you consider and work in accordance with your client's values and beliefs when implementing [intervention/s]?
When do you advocate for your clients?
How do you predict your clients will go according to [intervention/s]?
How do factors such as a client's social support and circumstances influence [intervention/s]?
How do you measure the outcomes of interventions with your clients?

### 2.4 Procedure

Two complementary methods were used to collect qualitative data based on the convenience and availability of participants: semistructured interviews were conducted with eight individual participants either face‐to‐face, via phone call, or video conferencing, and a focus group was conducted with four participants. The semi‐structured interview format allowed additional probing based on topics raised by participants. Data collection was conducted by one author (DT) and audio recorded following consent. Interviews lasted between 25 to 118 min (Mean = 50, SD = 24).

### 2.5 Data analysis

A modified template analysis approach was used, whereby the coding template was developed from a literature review, the application of a clinical reasoning model (Unsworth, 2017), and from coding a subset of transcripts (Brooks et al., 2015). First, a set of high order a priori codes on modes of reasoning were developed as an initial coding template. The use of a priori themes is advantageous when important theoretical concepts or perspectives have informed the aims of the study (Brooks & King, 2014). One author (DT) conducted the first round of analysis creating lower order codes (subthemes) from the sample of participants with more than 30 years' experience of occupational therapy (*n* = 4). These codes were refined in consultation with co‐authors and applied to the remainder of the transcripts with data saturation indicated. The final template is shown in Table 3. Upon completion of analysis, interpretations were sent to participants for member checking prior to inclusion in the study.

**TABLE 3:** Final coding template: themes and subthemes

Theme	Subtheme	Unsworth hierarchical model of reasoning (2017)
Ethical reasoning	Client independence vs. public safety Objectivity Advocacy	Higher order reasoning
Procedural (P) reasoning	Diagnostic implications (data collection) Cognition and insight (hypothesis testing) Grading the on‐road component of driver training	Middle level (including generalisation reasoning) P + I P + C C + I P + I + C
Interactive (I) reasoning	Forming partnerships with clients Trialing options Negotiating intervention procedures Liaison with driver instructors Liaison with other health professionals	
Conditional (C) reasoning	Driving is important (past, present, future) Therapist's role and boundaries (participation in therapy)	
Narrative reasoning	Life roles and occupational choices Family and social support	Middle level (including generalisation reasoning) Systematic, meaning making using a combination of biomedical and phenomenological approaches
Scientific reasoning	On‐road assessment Research Evidence, theoretical frameworks and guidelines Capacity to learn and modify driving performance	
Pragmatic reasoning	Funding and insurance Access Local road environment Licensing, transport authorities and vehicle modifiers	Basic level reasoning

## 3 FINDINGS

First‐level modes of clinical reasoning, were defined a priori per Template Analysis methodology, including diagnostic and procedural. Second‐level themes emerged from participant accounts articulating how the reasoning types integrate in occupational therapy driver rehabilitation intervention practice, shown in Table 3. Subthemes and a priori reasoning modes were cross referenced to identify combinations within the therapists reasoning. This section presents findings via the layers of the Unsworth (2017), excluding the therapists worldview which was not explored, to elucidate the contribution of each mode to the complexity of reasoning reflected in the transcripts.

### 3.1 Ethical reasoning

Three subthemes were identified under the mode of ethical reasoning: client independence versus public safety, objectivity, and advocacy. The aim of the occupational therapists work throughout driver rehabilitation was imbued with an overarching awareness of safety, of clients, and of other road users.

Everything around driving is the balance between promoting someone's independence as much as we can, but also maintaining safety in our broader community. (11)

Driving rehabilitation presented ethical dilemmas, the most salient of which was balancing a client's need for independence with the safety of the public. Occupational therapists perceived their role in driver rehabilitation as objective; thus, reserving judgment about a client's personality to focus on the outcomes of driving assessments, even when they “found a client personally challenging” (02). Objectivity also referred to managing closure of driver rehabilitation, by depersonalising unsuccessful outcomes, for example, “that their brain or body was letting them down rather than they had not put in the effort needed” (09). Advocacy was important for driver rehabilitation and for best client outcomes. Ethical tensions were resolved as calls for action in the interest of clients. Therapists advocated for their client with funders and other bodies to support clients where a return to driving was the best outcome for the client and their families.

### 3.2 Procedural reasoning

Three subthemes included the implications of diagnoses as found in data collection processes, the impacts of cognition and insight, and grading the complexity of the on‐road component of driver training. Diagnostic considerations were at the forefront of intervention planning when an illness was anticipated to deteriorate. Therapists adopted prognostic thinking, drawing from their body of knowledge and practice experiences to realistically estimate a client's remaining driving life and the amount of intervention required therefore reflecting conditional reasoning in action. Therapists also reported targeting specific driving related functions based on the client's diagnosis. For conditions that were expected to remain stable, the impact of a diagnosis on driving performance was nevertheless a key consideration. Occupational performance problems associated with a diagnosis were used as specific target areas in rehabilitation, for example, visual field for those with hemianopia or quadrantanopia.

If they've shown in the assessment they were able to take on board feedback and modify their behaviour, then that would be a cue for me that they've got potential for improving through rehab. Insight's a big one. If they have absolutely no insight into the errors that they're making, and think that they've done a great drive, then that's a bit of a red flag. (01)

Cognitive capacity was identified as having a more significant impact on likelihood of good driver rehabilitation outcomes than physical impairments. Clients needed to demonstrate a cognitive capacity to learn use of personal adaptations and vehicle modifications. There was a strong emphasis on observing client insight or awareness of errors during the on‐road assessment, as an indicator of rehabilitation potential. However, participants also noted that some clients had the insight to come to the decision not to pursue a return to driving. Grading the complexity of on‐road exposure during training was a crucial component of driver rehabilitation. By increasing traffic density, driver‐trained occupational therapists were able to observe the impacts of conditions such as changing terrain on driving performance.

### 3.3 Interactive reasoning

Five subthemes were identified from the data under the mode of interactive reasoning: forming partnerships with clients, trialing options, negotiating intervention procedures, liaison with driving instructors, and liaison with other health professionals as an additional form of interaction and connected to scientific and conditional reasoning. Throughout therapy, clients were encouraged to monitor their own progress. Occupational therapists gathered this information through interactive dialogues with their clients. Clients were supported to collaborate in therapeutic decision‐making by interactive prompting to reflect on their own goals and performance.

So I guess I will often use the process … to help them evaluate how much pain they're willing to put up with; how much they're willing to restrict their occupational performance with driving without a modification versus the modification, so it's shared decision making the whole way through. (06)

Driver rehabilitation options were explored with clients as a hierarchical process, with therapists interacting with their clients to determine which intervention type fits best. Therapists would start with more basic adaptive equipment before moving onto more complex equipment. Trialing options gave clients the ability to choose interventions, which contributed positively to uptake and adaptation to changes in driving mode or modifications. Rehabilitation procedures were negotiated with clients as a two‐way conversation. Participants emphasised the importance of using clear, simple language throughout this interaction, and where possible, explanations were kept specific to driving tasks and safety. Liaison with driving instructors was key to monitoring the progress of driver rehabilitation. Selecting the right instructor to match client needs was a considered process. However, sometimes best‐fit was not always possible due to the limited number of driving instructors, especially in regional areas. Participants sought advice from medical professionals regarding prognosis of a condition and likely impacts on driving performance. Medical professionals also helped with decision‐making about interventions, assisting a person come to terms with recommendations. Driver rehabilitation was regarded as a motivator for therapy in other areas, yet participants also asserted there was a clear distinction between driving and general rehabilitation.

### 3.4 Conditional reasoning

Four subthemes were identified under the mode of conditional reasoning: driving is important, therapist's role and boundaries, acceptance of interventions, and outcomes of interventions. Occupational therapists discussed a strong appreciation for the importance of driver rehabilitation, because driving enables participation in activities that give purpose and meaning to people's lives.

The benefits are really around what opportunities to provide that individual to be independent … when people drive, their health outcomes are better, they'll participate more. (11)

An appreciation of the detrimental impacts of driver cessation was also conveyed, with therapists aware that their recommendations may inform a decision to restrict a client's driver's license. Therapists appreciated that the loss of a driving license impacts on clients' sense of independence and autonomy, linking back to the overarching ethical stance of the therapists.

Cessation of driving is often going to lead to them become dependent on others for transport, it's going to reduce their capacity to engage … to increase the amount of social isolation. (05)

Occupational therapists' knowledge was used to help people with driver rehabilitation. However, limitations of their role were also identified in the data. Overall, participants recognised that their role intersects with that of other professional groups with differing expertise and that they had to stay within their own clinical knowledge and expertise and call on that of others as needed. Return to driving was undoubtedly the most apparent outcome for driver rehabilitation and was highlighted as a rewarding factor in their work. A new or renewed license had an enormous impact on a client's life, opening choices, and increasing access to their community.

### 3.5 Scientific reasoning

Three subthemes were identified: on‐road assessment; research evidence, theoretical frameworks, and guidelines; and a capacity to learn and modify driving performance.

The outcome of the on‐road driving assessment that preceded the rehabilitation phase was the strongest practice evidence described by participants, a form of hypothetic‐deductive reasoning that wove through responses. That is, it appeared difficult to speak of rehabilitation planning without reflecting on assessment results, from the pre‐on road and continued observations of performance. As such, the range of assessments conducted by the driver‐trained occupational therapists routinely bore the heaviest weight in their overall decision‐making. However, any hypothesis built on clients' self‐evaluation, overall presentation, or off‐road assessment were ultimately superseded by a client's performance on‐road. Therapists reported not making any final decisions about rehabilitation until the client's on‐road performance had been observed.

I want to see that [skill] consistently over a number of lessons. I don't want to want to see it once in one lesson, I want to see it every time that they needed to be doing that. (06)

Participants asserted that peer‐reviewed scientific research evidence for particular driver rehabilitation interventions was sparse, so they often deferred to clinical judgment and experience to guide practice. In the absence of specific driver rehabilitation intervention research evidence, general evidence was transferred across to a driver rehabilitation context, applied and then evaluated. Clients had to consistently demonstrate their capacity to learn and modify their driving performance. Driver‐trained occupational therapists tested hypotheses about the stability of a client's learning progress over several occasions to ensure that modified driving skills had been embedded.

### 3.6 Narrative reasoning

Two subthemes were identified under the mode of narrative reasoning: life roles and occupational choices, and family and social support. Life roles and occupational choices had a considerable impact on driver rehabilitation, including the timing and urgency of implementation. Understanding a person's life roles supported shared decision‐making and improved communication. For example, if a client needed to return to driving urgently due to family circumstances, a modification might be selected to facilitate a quicker return to driving.

She really does need to get back to driving, because her husband's about to start chemotherapy. And we say to her, “Look, quite honestly, in our opinion, you're going to get back quicker if you use a spinner knob.” (08)

Furthermore, occupational therapists placed higher driving performance expectations on clients who worked with motor vehicles or construction equipment, especially if this involved transporting other people, again reflecting the ethical stance of public safety. A return to work was highlighted as a strong, motivating factor for the effort required for driver rehabilitation, facilitated by external funding incentivisation. Support from family was important and could facilitate or “sabotage a return to driving” (05). Family members could be “brought on board with a return to driving by being invited into the car for a treatment session to observe safe driving behavior with or without modifications” (07). Conversely, the presence of modifications on the family vehicle could be discouraged by other car users. Family members played a key role in maintaining a client's community mobility which was facilitated by three‐way conversations between the client, family member and occupational therapist.

### 3.7 Pragmatic reasoning

Four subthemes were found under the mode of pragmatic reasoning: funding and insurance, access, local road environment and licensing, transport authorities, and vehicle modifiers. Driver rehabilitation interventions become more expensive with complexity. Occupational therapists used funding to determine realistic recommendations about what each client can afford.

I've got a client at the moment who needs to have hand controls. She's a learner driver, but they don't have a vehicle that hand controls can be fitted into, and they're not in the financial position to purchase a vehicle … All of that goes into my recommendations. (04)

Occupational therapists viewed themselves as a buffer between clients and funding providers, including the National Disability Insurance Scheme (NDIS), which reportedly presented challenges for resource access and decision‐making. Access to the vehicle and modifications was essential for rehabilitation, and where a client lived impacted access to services. Participants had to consider access to a suitable vehicle and modifications alongside other factors such as client needs, funding levels, and support. A client's capacity to negotiate all aspects of their local road environment was essential. Occupational therapists reported using a variety of road environments and driving tasks to assess and extend client skills and endurance. Negotiating and working with licensing authorities was part of the process of a return to driving. These processes were often complex, and the participants sought to guide their clients through these external processes to achieve the desired outcome.

In sum, the therapists in the study integrated all modes of reasoning in their roles in driver assessment and rehabilitation. Participants told interwoven stories of complex, ethically challenging interactions with clients and families, other health professionals and systems. The following quote by an occupational therapist with 35 years' experience, 15 in driver rehabilitation, illustrates blended modes of reasonings that in the Unsworth Hierarchy would be represented as generalisation reasoning frame (see Table 3)

A woman I did a driver assessment with last week for a left‐foot accelerator. So, she's only been able to drive about, fifteen or twenty minutes. She wants to visit her mum … which is almost a two‐hour drive, but the only way she can visit her at the moment is with her husband driving her there and back; the left‐foot accelerator not only reduces her pain experience significantly, but it probably means it's going to be able to get her to a situation where she can visit her mum independently, was such a motivator, so, I guess having that information about someone, and that story; to know that is important to her, really helps in explaining process and the benefits of it and for her, her willingness to uptake that intervention, and that modification (6)

## 4 DISCUSSION

This study elucidated the professional reasoning applied in practice by 12 driver‐trained occupational therapists of various experience levels from across Australia when implementing rehabilitative interventions. In seeking to explore their reasoning, therapists were able to narrate and exemplify their practice, reflecting on the competencies for driver‐trained occupational therapists (Fields et al., 2018). Unsurprisingly, the participants never explicitly stated the mode of reasoning they used, but when narratives were combined, a coherent and stable pattern of assessment and intervention strategy emerged. Modes reflected the hierarchy of reasoning and included ethical, procedural, interactive, conditional, narrative, scientific, and pragmatic, and all forms were evident in each of the participants interviews. No new forms of clinical reasoning appeared, indicating the existing theory described in the wider occupational therapy literature is sufficient to capture the practices of complex interventions in occupational therapy, such as driver rehabilitation.

Although template analysis uses a priori concepts, the analytical structure remains tentative enough to allow new or unidentifiable codes to emerge (Brooks et al., 2015), but this did not occur for completely new reasoning modes. However, an interesting coding dilemma occurred in the findings, regarding the interactivity described by participants with key health and licensing stakeholders in the driver assessment and rehabilitation process. This study analysis coded these findings with interactive reasoning, also linking it to scientific and conditional modes, although Unsworth and other theorists typically describe interactivity as a therapist to client endeavour. The profession‐to‐profession interaction might be coded to diagnostic reasoning, which is usually described as a subset of or procedural reasoning. This interactivity is a clinical exploration and communication method by which the rehabilitation interventions and decisions are mediated, and as such exemplify the integrated nature of reasoning itself and complexity of delineating modes.

Acknowledging the complexity of clarifying and describing reasoning in action, particularly in an area of advanced scope of practice, the findings suggest modes of reasoning connected to certain aspects of the rehabilitative process or areas of therapist concern. For example, when considering a person's cognitive capacity and insight, scientific and conditional reasoning modes were most apparent, indicating the therapist moving between mid‐levels of the reasoning hierarchy. However, therapists also described safety versus independence as an overarching confirmation, suggesting higher order ethical reasoning was also present. Alternatively, when considering the local road environment, ethical plus interactive, and pragmatic reasoning modes dominated, indicating all levels of the Unsworth (2017) hierarchy in practice simultaneously. When liaison with family occurred, narrative reasoning was added to the use of ethical, interactive, and pragmatic, further thickening the layers of the reasoning. In choosing the most helpful driving instructor, conditional and pragmatic reasoning was added to the modes in use. When interacting with that driving instructor to maximise client outcomes in rehabilitation, other modes became apparent. When considering access issues, pragmatic reasoning was most prominent. Across the findings, the most applied mode of reasoning appeared to be interactive, perhaps reflecting the value set of client‐centeredness in occupational therapy practice (Fearing & Clark, 2000).

Interview questions in this study were designed so that participants had scope to explore their reasoning, with a priori consideration of modes but without specifically using the terms in the questions themselves. There was no obvious pattern to which mode was used in accordance with questions however, and it was observed that participants elaborated on their own responses, linking and building on different modes of reasoning as they narrated their practice. That is, the interview might start with a question that explored procedural reasoning, but the participant might start their narrative with a contextual and ethical standpoint. Unsworth's (2011) case study of clinical reasoning in on‐road driver assessment also found all modes of reasoning, identifying that procedural reasoning was consistently present. In this study, while procedural reasoning was evident, it did not dominate, rather ethical, interactive, conditional, narrative, and pragmatic reasoning modes appeared more evident. Comparison of these findings reflect two important aspects of professional reasoning theory, including that the reasoning of advanced practitioners is hypothesised to be less procedural (O'Brien, 2018) but also that therapists who undertake driving assessment and rehabilitation shift may from a more consistent procedural mode during one phase (assessment) and into more use of the hierarchy of modes during the rehabilitation phase. However, the results also indicate evaluation (usually called assessment by the therapists) is ongoing throughout driver rehabilitation. The mode shifting observed here is likely mediated by the demand of the therapist's role, or as Sinclair (cited in Cole & Creek, 2016) states, “occupational therapists employ different modes for different tasks and can develop expertise in some while remaining novice in others” (p 15). The advanced practice driver‐trained occupational therapist appears to be skilled across most domains and able to mode shift.

This study has illustrated the driver‐trained occupational therapist uses the standpoint of safety versus client independence as a shared ethical orientation to the role and then integrates all other modes into their decision making and communication when designing a rehabilitation programme and implementing it with their clients. Thus, confirming previous work that modes of reasoning are rarely used in isolation and that the process of clinical reasoning is not linear (Unsworth, 2017). For example, a question about a client's diagnosis might espouse an ethical dilemma, which then might impact interactions with a driving instructor—within the context of how important driving is to a person. Further, advanced scope therapists operating in this multi‐modal way may have reached a level where clinical intuition is used, “defined as knowledge that is immediate and accessed without a conscious awareness of reasoning” (Chaffey et al., 2010 p. 88). The challenge to the experienced therapist, particularly when coaching or mentoring a novice or advanced beginner, is to elucidate and bring to consciousness the tacit knowledge and skills, without “diminishing it to oversimplified lists of actions, without the context in which they are used” (Hagedorn 2000, cited in Cole & Creek, 2016). These findings are pertinent to the postgraduate education of driver assessment and rehabilitation occupational therapists, where typically advanced scope therapists are training the less experienced. For example, the iterative and complex nature of the therapists reasoning could be described explicitly, with an emphasis on the conditional and ethical modes of reasoning developed from experience, in addition to competency‐based and procedural aspects of the role which are a strength of current educational offerings.

### 4.1 Limitations

Participants in this study were primarily experts in their field of practice, with the fluidity of their reasoning modes indicative of years of experience. The driver assessment and rehabilitation competencies for practice are relatively stable across jurisdictions; however, institutional contexts may have influenced the findings somewhat in the pragmatic domains. The use of template analysis was a novel approach to the study, but application of qualitative inductive analysis may have yielded different patterns of findings. It was observed that each participant built upon and expanded their own reasoning narrative in response to the interview prompts, whereas the utilisation of a focus group introduced another dimension of reflective opportunity as participants were observed to build upon and clarify each other's responses in addition to their own. However, it is unknown if focus group participants felt limited by the presence of others. Group data collection appears to create depth of responses, but future research might consider means to offer reflective opportunity whilst controlling for possible group effects. The therapists' personal world view and its relationship to reasoning in this area of practice could be explored in future research.

## 5 CONCLUSION

The study was designed to explore how driver‐trained occupational therapists use clinical reasoning to develop and implement complex driver rehabilitation programmes. The therapists used practice‐based narratives, describing their ethical stance and a shared fluidity of hypothesis testing, observation of performance, interactivity, and conditional and pragmatic decision making. These findings respond to a recommendation of Marquez‐Alvarez et al. (2019) for more research into modes of reasoning. The findings support the view of driver rehabilitation as an advanced scope of practice as the therapists demonstrated higher order, mid and lower‐level hierarchical reasoning across the domains of their practice endeavours. The research did not aim to create a definitive guideline but can support occupational therapists to reflect upon their clinical reasoning when moving between skills sets within driver assessment and rehabilitation service provision, and when changing scope of practice to commence occupational therapy driver assessment and rehabilitation training and practice.

## CONFLICT OF INTEREST

The authors have no conflicts of interest to declare.

## AUTHOR CONTRIBUTION

All authors contributed significantly and are in agreement of the content of the manuscript. The study was conceived and designed by all authors. Author 3 completed data collection, with author 2 being instrumental in analysis.
